# Intraepithelial lymphocytes in the pig intestine: T cell and innate lymphoid cell contributions to intestinal barrier immunity

**DOI:** 10.3389/fimmu.2022.1048708

**Published:** 2022-12-08

**Authors:** Jayne E. Wiarda, Crystal L. Loving

**Affiliations:** ^1^ Food Safety and Enteric Pathogens Research Unit, National Animal Disease Center, Agricultural Research Service, United States Department of Agriculture, Ames, IA, United States; ^2^ Immunobiology Graduate Program, Iowa State University, Ames, IA, United States; ^3^ Department of Veterinary Microbiology and Preventative Medicine, College of Veterinary Medicine, Iowa State University, Ames, IA, United States

**Keywords:** porcine, biomedical model, intestinal lymphocyte, swine, epithelial barrier, intestinal epithelium, T lymphocyte, innate lymphoid cell 1

## Abstract

Intraepithelial lymphocytes (IELs) include T cells and innate lymphoid cells that are important mediators of intestinal immunity and barrier defense, yet most knowledge of IELs is derived from the study of humans and rodent models. Pigs are an important global food source and promising biomedical model, yet relatively little is known about IELs in the porcine intestine, especially during formative ages of intestinal development. Due to the biological significance of IELs, global importance of pig health, and potential of early life events to influence IELs, we collate current knowledge of porcine IEL functional and phenotypic maturation in the context of the developing intestinal tract and outline areas where further research is needed. Based on available findings, we formulate probable implications of IELs on intestinal and overall health outcomes and highlight key findings in relation to human IELs to emphasize potential applicability of pigs as a biomedical model for intestinal IEL research. Review of current literature suggests the study of porcine intestinal IELs as an exciting research frontier with dual application for betterment of animal and human health.

## Introduction

Intestinal intraepithelial lymphocytes (IELs) are “lymphoid cells that reside between the intestinal epithelial cells (IECs) that form the intestinal mucosal barrier” and include subsets of both intraepithelial T lymphocytes (T-IELs) and intraepithelial innate lymphoid cells [ILC-IELs (1);]. Close physical proximity to the intestinal lumen lends IELs the unique task of serving as first line immune mediators, requiring balance between immune defense versus tolerance towards enteric microbes and dietary substances. IELs are most well-studied in humans and rodent models, where roles in wound healing (2, 3), tissue homeostasis (3–5), epithelial surveillance (6, 7), epithelial integrity (7, 8), epithelial cell shedding (9), nutrient sensing (10), immune regulation (3, 11), pathogen defense (7, 12, 13), intestinal inflammation (14–16), and immunopathology (17–20) have been demonstrated and reviewed extensively (1, 21–29). IELs are also identified in veterinary species, including in porcine (referenced throughout), bovine (30–32), ovine (33), caprine (34), camelid (35), equine (36, 37), and avian (38–40) intestine. However, the biological significance of IELs across different species is not fully delineated, as veterinary research pertaining to IELs trails far behind that performed in humans and rodents.

Pork is the most highly consumed animal protein in the world (41), and keeping pigs healthy is crucial for global food security. Unlike humans, pigs are born without maternally-acquired passive immunity due to placental differences (42). Instead, piglets must ingest colostrum to acquire most maternally-derived immune factors. Piglet rearing environment can also be an especially formative influence for immune education and immune system development in early life (43–45) and is very different from humans. Conventional production pigs in developed countries are weaned at a relatively early age while intestinal immune development is still ongoing (46), predisposing pigs to adverse early-life events that may be detrimental to gut health and might shape the long-term intestinal immune landscape (47, 48). Hence, understanding how intestinal immune components, including IELs, are shaped by early life events is crucial for identifying processes promoting intestinal health over dysregulation. In addition, delineating trajectories of how intestinal development and cellular maturation might be altered through targeted intervention strategies is a cutting-edge approach for promoting a healthy gut (discussed in subsequent sections). In particular, immunomodulation of IELs at the intestine-lumen interface is a potential targeted strategy to maximize intestinal health without the use of antibiotics. All in all, the biological significance of IELs, global importance of pig health, and potential of early life events to influence how IELs impact intestinal and overall pig health point to a necessity for better biological understandings of IELs in the developing porcine intestinal tract.

This review reiterates current knowledge of porcine IELs, pertaining to dynamics of functional and phenotypic maturation in the context of the developing pig gut. Based on available findings, we formulate probable implications of IELs on intestinal and overall health outcomes as the intestinal immune system develops in the growing pig. We also highlight key findings in relation to human IELs, emphasizing the advantages and potential drawbacks of pigs as biomedical models for intestinal IEL research. In total, findings present porcine intestinal IELs as an exciting research frontier with application to both pig and human health.

### IEL identification in pig intestinal tissues

While the root definition of IELs is based on location within the epithelium (situated between the epithelial basement membrane and the luminal surface), IEL distribution throughout the intestinal tract and/or within different regions of the epithelium can provide further insight into cellular function and biological importance. Thus, it’s integral we are able to identify IELs within their native tissue structures through histological examination of IELs with various *in situ* staining techniques.

#### Hematoxylin and eosin (H&E) staining identifies IELs

**Figure 1:**
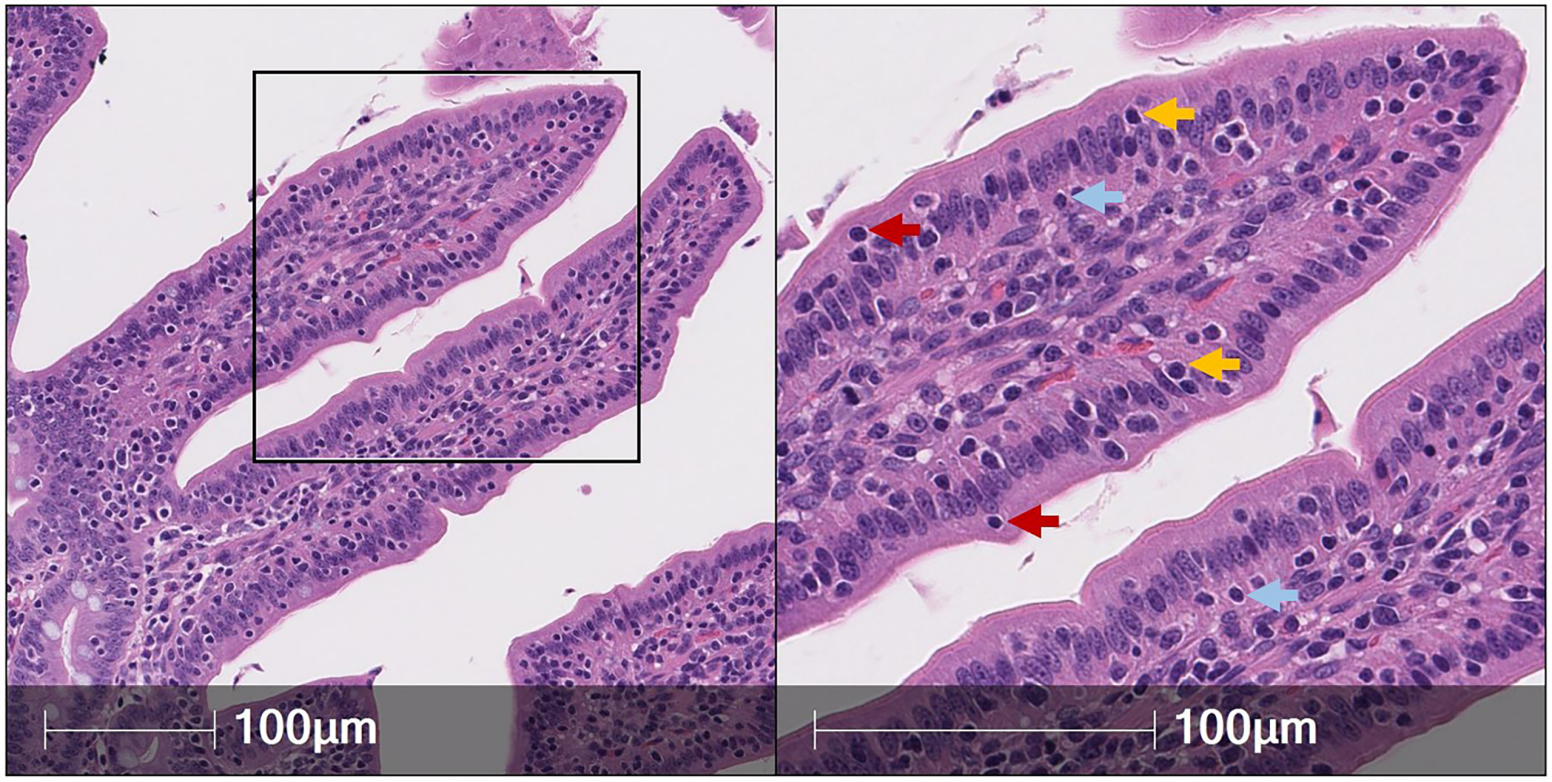
Identification of IELs from H&E staining in pig intestine. H&E-stained tissue section of jejunum from a ~9-week-old pig. IELs are identified as dark purple, round nuclei within the epithelial layer. Two each of IELs in apical epithelium (red arrows), nuclear-level epithelium (gold arrows), and basement membrane (light blue arrows) are indicated in the panel on the right. H&E (hematoxylin and eosin); IEL (intraepithelial lymphocyte).

Lymphocytes subjected to H&E staining are customarily small cells (~7-um diameter) mainly composed of large, dark, rounded nuclei (stained with hematoxylin to identify acidic components in purple) and minimal cytoplasm [stained with eosin to identify basic components in pink (49)]. Therefore, IELs can be distinguished from IECs based on nuclear and cytoplasmic H&E staining properties [**Figure 1** (50)]. However, a major drawback of IEL identification *via* H&E staining is the inability to further differentiate IEL subsets (e.g. T-IELs, ILC-IELs). As demonstrated in humans, IEL quantification *via* immunohistochemistry (IHC) has increased sensitivity compared to H&E quantification, even when staining for protein markers that only recognize subsets of IELs (51, 52). Thus, H&E identification of IELs in pigs could also potentially have decreased sensitivity compared to additional *in situ* staining methods discussed below.

#### Identifying IELs *via* immunohistochemical detection of CD45, CD3ε, and CD2 proteins

Porcine IELs can be identified by IHC using antibodies to label cluster of differentiation (CD) proteins expressed on the cell surface of lymphocytes within the epithelium. In pigs, a definitive pan-IEL protein marker has not been established, though the majority of porcine IELs appear to express CD45, CD3ε, and CD2 proteins when labeled *in situ* (as described below).

CD45 is a phosphatase that regulates cellular signaling/activation [reviewed in (53, 54)] and is considered a pan-leukocyte marker expressed by >95% of circulating lymphocytes in pigs (55, 56). Though the percentage of CD45^+^ intestinal IELs in pigs has not been quantified to our knowledge, most IELs are likely CD45^+^ (similar to circulating lymphocytes). However, non-lymphocytes in the epithelial layer, such as eosinophils and dendritic cells (DCs), likely also express CD45 (55, 56), limiting the ability of CD45 to serve as a marker for only lymphocytes. We’ve established CD45 as an unsuitable pan-innate lymphoid cell (ILC) marker in the pig intestine, as *PTPRC* (gene encoding CD45) has decreased expression in some intestinal ILC populations, and not all intestinal ILCs express CD45 protein (57).

CD3ε is an accessory molecule to the T cell receptor (TCR) and is considered a pan-T cell marker in pigs (55, 58), unlike additional TCR accessory molecules, CD3δ, CD3γ, and CD3ζ, that are detected in both porcine T cells and ILCs at the transcriptional level (57, 59). On the other hand, CD2 is a cell adhesion molecule and co-stimulatory receptor that is expressed by both T cells and ILCs in pigs, humans, and mice (55, 58–61). Therefore, CD3ε can be used to detect T-IELs but not ILC-IELs in pigs, while CD2 might serve as a reliable marker to identify both T-IELs and ILC-IELs in the intestinal epithelium. However, a small subset of γδTCR T cells in the porcine intestinal tract are CD2^-^ (62), leaving the potential of some intestinal T-IELs being CD2^-^ in pigs. Identification of CD2^-^ γδTCR T cells in the porcine intestine by staining with antibody clone MAC320 [reactive to swine workshop cluster 6 (SWC6) protein expressed by porcine CD2^-^ γδTCR T cells (63, 64); to-date, SWC6 protein has not been fully characterized] demonstrated very few SWC6^+^ cells (inferred to be CD2^-^ γδTCR T cells) present, and none were detected in the epithelium (65). In addition, there are very few CD2^-^ γδTCR T cells in small or large intestinal epithelium (66, 67), confirming CD2^-^ γδTCR T-IEL absence or rarity throughout the porcine intestine. Therefore, CD2 still remains a potential marker for identifying the majority of IELs in pigs.

Previous *in situ* work found most but not all CD2 staining colocalizes with CD3ε staining (68). Moreover, recent work identified CD2^+^CD3ε^-^ cells in porcine ileum (57), suggesting the presence of CD2^+^CD3ε^-^ ILC-IELs. Collectively, CD3ε appears to be a reliable marker for detecting T-IELs, while CD2 remains a more reliable *in situ* marker to encompass T-IEL and ILC-IEL detection at the protein level, though additional biological contexts (e.g. age, disease state) warrant further exploration for more comprehensive validation.

#### Utilizing RNA *in situ* hybridization (RNA-ISH) to further delineate IEL populations

mRNA-reactive reagents can more easily be synthesized and optimized for *in situ* detection than can antibodies reactive to targeted proteins (69). Given the paucity of porcine-specific immunoreagents, RNA-ISH has become an important tool for identifying and characterizing porcine cell types. Recent work documents discovery of ILC-IELs in the porcine ileum based on dual labeling for *ITGAE* mRNA [encoding the integrin α subunit of CD103 (aka α_E_β_7_), considered a pan-marker of IELs and tissue residency in humans and mice (70–72)] and CD3ε protein [a porcine T cell marker (55, 58)]. Based on dual *ITGAE/*CD3ε staining, ILC-IELs are identified as *ITGAE* ^+^CD3ε^-^ cells, while T-IELs are identified as *ITGAE* ^+^CD3ε^+^ cells (57). Based on transcriptional properties, ILC-IELs are further classified as group 1 ILCs, which includes both ILC1s and natural killer (NK) cells. However, group 1 ILCs in the porcine intestine differ transcriptionally from NK cells, which are infrequent in the porcine intestinal tract (57), thus suggesting a non-NK cell identity for porcine intestinal ILC-IELs. Since *ITGAE* is expressed by both CD3ε^+^ and CD3ε^-^ cells within the epithelial layer of porcine ileum, *ITGAE* might be a pan-IEL marker for both T cells and ILCs in pigs. *ITGAE* ^+^CD3ε^+^ vastly outnumber *ITGAE* ^+^CD3ε^-^ IELs in the ileal epithelium of 7.5-week-old pigs (57), indicating T-IELs (*ITGAE* ^+^CD3ε^+^) are more abundant than ILC-IELs (*ITGAE* ^+^CD3ε^-^) in pigs at the only age and intestinal location thus far analyzed. Findings are similar to humans, where >90% of intestinal IELs are T-IELs (1), but further probing into additional ages, intestinal locations, and biological contexts in pigs remains to be studied.

**Figure 2:**
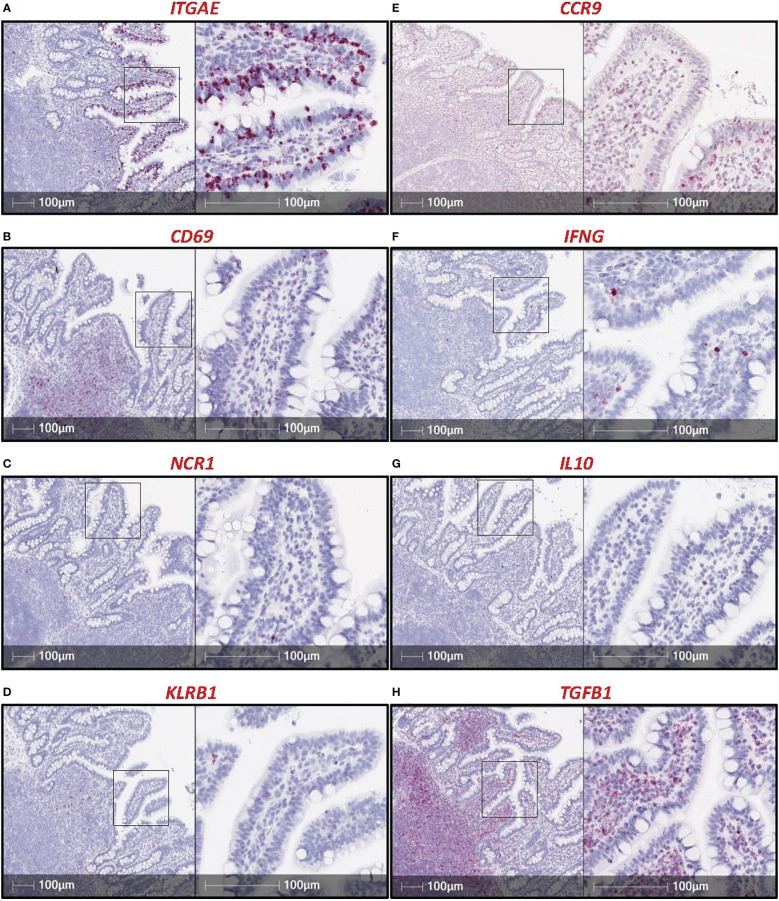
RNA-ISH staining of potential IEL markers in porcine ileum. Sections of ileum from a ~7.5-week-old pig stained for mRNA transcripts (red staining): *ITGAE* **(A)**, *CD69* **(B)**, *NCR1* **(C)***, CD161* **(D)***, CCR9* **(E)***, IFNG* **(F)***, IL10* **(G)***, TGFB1* **(H)**. Probes were custom-designed from Advanced Cell Diagnostics with the following catalog numbers: *ITGAE* (#590611); *CD69* (#590601); *NCR1* (#553131); *KLRB1* (#490871, product labeled CD161); *CCR9* (#1062631); *IFNG* (#490821); *IL10* (#442791); *TGFB1* (#444951). A protocol for RNA-ISH staining is described previously (57). RNA-ISH (RNA *in situ* hybridization).

CD69, a marker associated with a tissue resident phenotype within the epithelial layer, is used to identify IELs in other species (1). Our lab investigated the potential use of *CD69* transcript as another IEL marker in pigs but determined, unlike *ITGAE*, *CD69* is not largely expressed by cells in the epithelial layer of the porcine intestinal tract (**Figures 2A,B**). Additional markers with known expression by IELs in other species include *NCR1* [encoding NKp46 (73)], *KLRB1* [encoding CD161 (74)], and *CCR9* (75), all of which did not appear to be appropriate pan-IEL markers in pigs due to low/sporadic expression in the epithelium (**Figures 2C–E**). RNA-ISH detection of cytokine-encoding genes *IFNG, IL10*, and *TGFB1* were also tested, but positive staining was sporadic or non-specific to IELs (**Figures 2F–H**). Of mRNA targets evaluated thus far *via* RNA-ISH, only *ITGAE* presents as a promising pan-IEL gene marker to capture both T-IELs and ILC-IELs that likely encompass the total IEL community in the intestinal epithelium of pigs. However, CD103^+^ DCs in the epithelial layer (intraepithelial DCs; not reported in pigs thus far) would likely express *ITGAE* as well. Therefore, further methods to differentiate between *ITGAE^+^* lymphocytes and non-lymphocytes may be beneficial for more accurate identification of porcine IELs at the mRNA level, but reliable *in situ* markers for many non-lymphocyte immune cells also remain to be developed in pigs.

#### *In situ* identification of T-IEL subsets

Porcine T cells are further broken down into subsets of CD4 αβTCR T cells, CD8 αβTCR T cells, and γδTCR T cells based on expression of different cell surface molecules. Consequently, it has been attempted to differentiate between CD4 αβTCR, CD8 αβTCR, and γδTCR T-IEL subsets through *in situ* staining for CD4 and CD8α proteins. CD4^+^ cells are found in the intestinal lamina propria and surrounding Peyer’s patches but are completely absent or extremely rare in the intestinal epithelium of pigs (76–80), indicating CD4 αβTCR T cells are not major contributors to the IEL community in the pig intestine. CD8α^+^ cells are localized preferentially to the epithelium (76, 78–81), but porcine αβTCR T , γδTCR T, and NK cells can all express CD8α (58). While NK cells (CD3ε^-^CD8α^+^) are not highly prevalent in the porcine intestinal tract, both γδTCR and CD8 αβTCR T cells occur in the intestine of pigs at regular frequencies (62), and most γδTCR T-IELs are CD8α^+^ (66, 67). Thus, CD8α^+^ IELs mostly includes both γδTCR and αβTCR lineage T cells. A protocol for *in situ* detection of γδTCR-associated protein is required to detect γδTCR T-IELs but has not been established in pigs to our knowledge, and thus porcine γδTCR T cells have not been exclusively identified *in situ* at the protein level. Previously documented attempts for *in situ* detection of porcine γδTCR T cells included staining for SWC6 protein to detect CD2^-^ γδTCR T cells (65) and use of an anti-mouse γδ antibody with unverified reactivity in pigs (82).

**Figure 3:**
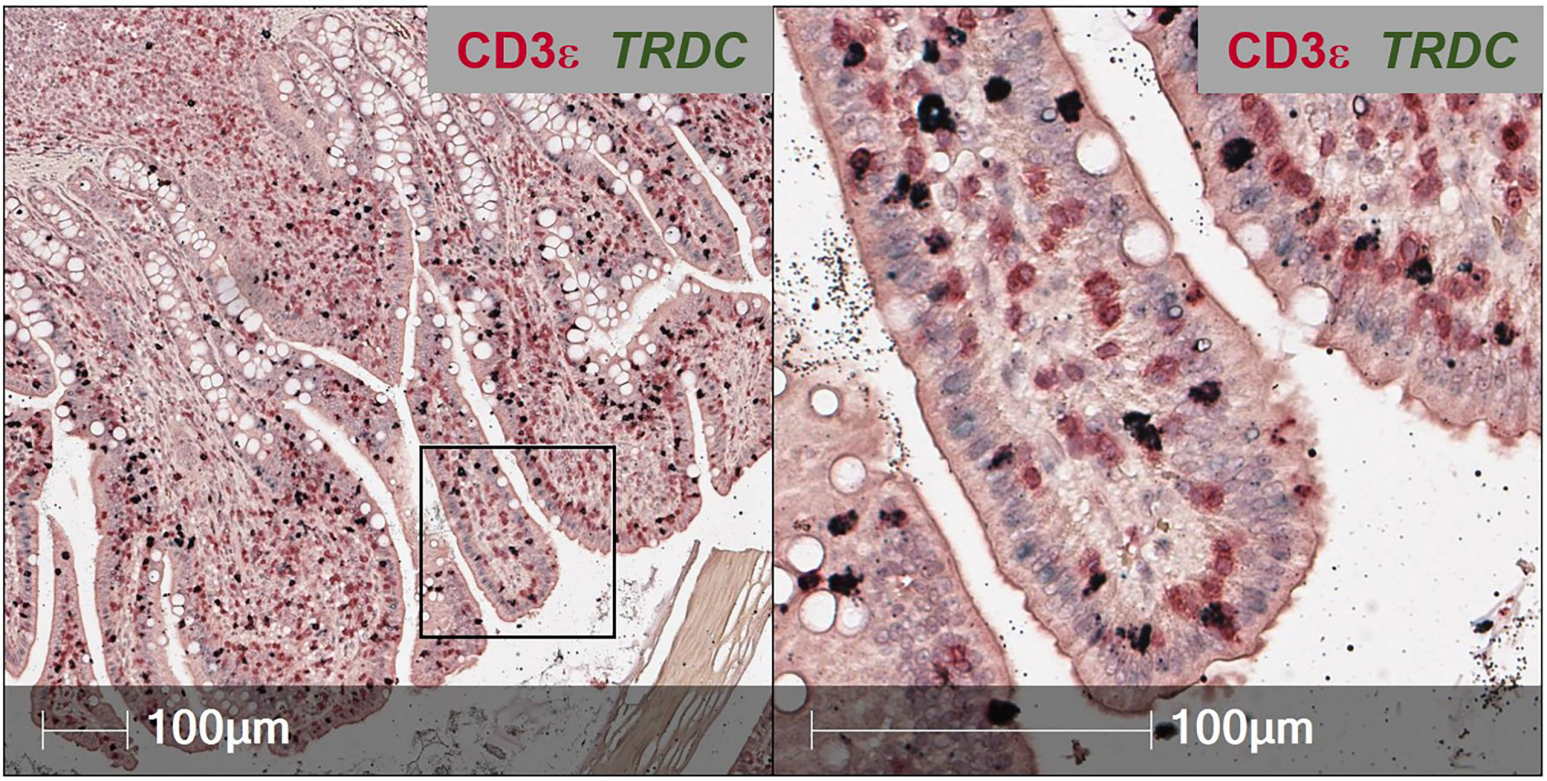
Dual *in situ* detection of αβ and γδ T cells in pig intestine. Section of ileum from a ~6-week-old pig stained for CD3ε protein (red) and *TRDC* transcript (dark green). *TRDC* ^+^ cells (dark green) are identified as γδ T cells, while *TRDC* ^-^CD3e^+^ cells (red) are identified as αβ T cells. The staining protocol was further optimized from previous work (57, 66) and is available at dx.doi.org/10.17504/protocols.io.q26g7yro1gwz/v1.

To overcome issues of αβTCR versus γδTCR T cell differentiation in pigs, a dual staining method was established (combining IHC and RNA-ISH), where IELs expressing *TRDC* (encoding the δ constant chain of the γδTCR) are identified as γδTCR T cells, and *TRDC* ^-^ IELs expressing CD3ε protein (*TRDC* ^-^CD3ε^+^) are inferred as αβTCR T cells (**Figure 3**). Using this method, γδTCR T cells were identified in both the small and large intestine and were mainly concentrated to the epithelium, indicating most intestinal γδTCR T cells are intraepithelial in pigs (66). Moreover, RNA-ISH staining for *CD4* and *CD8B* transcripts to detect CD4 αβTCR and CD8 αβTCR T cells, respectively, further supports *in situ* protein staining, showing *CD4* is not expressed in the epithelium, while *CD8B* staining is present in the epithelium (57). Unlike the protein marker CD8α, the RNA marker *CD8B* is exclusive to CD8 αβTCR T cells, as *CD8B* is not expressed in transcriptomes of ILCs (including NK cells), CD4 αβTCR T cells, or γδTCR T cells in pigs (57, 59). Collectively, IHC and RNA-ISH staining support ILCs, γδ TCR T cells, and CD8 αβTCR T cells preferentially located in the intestinal epithelium of pigs, while CD4 αβTCR T cells are sub-epithelial.

### Abundance and distribution of IELs throughout the intestine

To understand how IEL communities mature as the porcine intestinal tract develops, a baseline for IEL distribution within the intestinal tract of aging, healthy, conventional pigs must be established. Newborn pigs have very few IELs in small intestinal villi (65, 77, 79, 80, 83). At birth, only 38% of CD2^+^ cells are intraepithelial but increase to >50% in jejunum and >60% in ileum by seven weeks of age (77). In jejunum and ileum, total IEL numbers increase from ~2 cells per 100 enterocytes near birth to between 20 and 30 cells by nine weeks of age (65). Results thus indicate preferential localization of CD2^+^ cells in epithelium over lamina propria and expansion and/or recruitment of the intestinal IEL compartment with age.

IEL distributions within conventional, age-matched pigs differ by regional locality across proximal versus distal intestinal locations. In the large intestine, between 3.5-4 times as many CD3ε^+^ cells are located within the epithelium compared to lamina propria (84), while in the small intestine, approximately equal numbers of CD2^+^ lymphocytes are distributed between the epithelium and lamina propria (76, 77, 79). Though measurements of CD2^+^ and CD3ε^+^ cells are not necessarily equivalent, comparison suggests more preferential localization of lymphocytes to the epithelium of the large compared to small intestine. However, overall IEL abundance is greater in the porcine small than large intestine (66, 67, 85–90).

Though the largest differences in IEL distributions occur when comparing small versus large intestinal locations, comparisons between locations within small intestine or within large intestine also reveal important distinctions. However, study of porcine IELs has been largely focused to the small intestine, with very few comparisons of IEL distributions between large intestinal locations. In the large intestine, IELs are more abundant in cecum than in colon [per 100 colonocytes or percentage surface area (66, 91)]. In small intestine, IEL abundance across proximal and distal locations differ across studies; however, quantification of IELs are made with different methods that yield varying results where intestinal morphology need be considered. Quantification of total CD2^+^ intraepithelial cells per villus revealed greater IEL numbers in proximal over distal small intestine (76). Quantification of total CD3ε^+^ intraepithelial cells per villus revealed greater IEL numbers in jejunum over duodenum and ileum (87). Conversely, quantification of total IELs per 100 enterocytes revealed approximately equal or higher numbers in distal compared to proximal small intestine (65, 88, 90, 92–95), as did quantifying the number of CD2^+^ intraepithelial cells per a set number of microscopic fields (79) or the percentage of CD3ε^+^-stained intraepithelial surface area per villi (66). Coupled with knowledge that epithelial area per villus is greater in the proximal versus distal small intestine (96), results suggest more IELs are present in each of the larger villi of the proximal small intestine, such as the jejunum, but IELs in the distal small intestine, such as the ileum, are more concentrated within smaller villi. Additional biological and/or technical factors may also contribute to the variable findings across studies and might be indicative of functional roles across the intestinal tract yet to be understood.

IEL distributions also vary by locality within crypts and villi and in relation to luminal proximity, including location in the basement membrane, enterocyte nuclear level, or apical epithelium (**Figure 1**). In porcine small intestine, IELs preferentially congregate to intestinal villi over crypts at up to a four-to-one ratio (65–67, 77, 78, 83, 97, 98). Preferential accumulation of IELs in villi suggests IEL effector functions may involve close interactions with signals derived from the intestinal lumen, as villi are more intimately associated with the lumen compared to shielded niches of crypts. Similarly, reduced numbers of IELs in large compared to small intestine may be due to lack of luminal signals occurring in large intestinal crypts, supported by preferential congregation of large intestinal IELs to the upper third of the crypt that is in closest proximity to the lumen (84). Reduced numbers of IELs in the large intestine might also be attributed to differences in mucosal barriers, such as a double mucus layer providing enhanced separation between lumen and epithelium in the large intestine compared to only a single mucus layer in the small intestine (99, 100). IELs in small intestinal villi also differ in distribution across the epithelial layer in relation to luminal proximity. While the majority of IELs are located near the basement membrane, some IELs are in closer proximity to the lumen, including at the enterocyte nuclear level, apically, or even freely within the lumen (65, 67, 78, 79, 87, 97, 101). However, IEL detection within the free lumen must be cautiously interpreted, as technical variables of tissue processing may compromise tissue integrity. IELs in closer proximity to the lumen vary in phenotypes and potential effector functions compared to those located at the basement membrane, as is discussed in later sections of this review.

### IEL isolation for *ex vivo* phenotyping *via* flow cytometry

**Figure 4:**
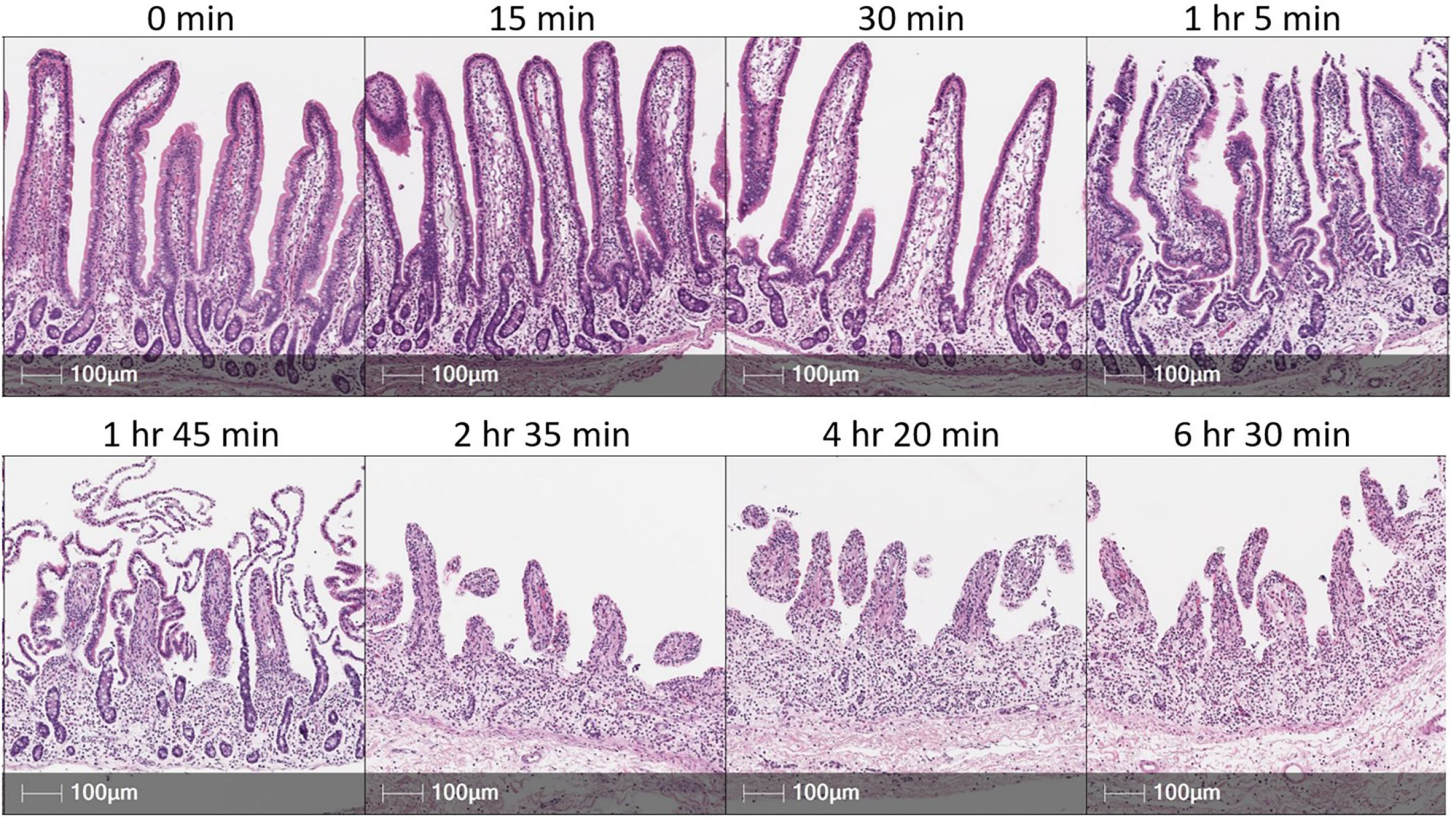
EDTA incubation time influences epithelial cell removal in porcine intestinal tissues. H&E sections of porcine small intestinal tissue subjected to incubation in EDTA solution for various amounts of time. Tissue was derived from terminal ileum of a ~3-month-old pig. After dissociating mucus from tissue [described elsewhere (66)], ~2 grams of tissue was placed into 200 mL of epithelial removal solution (5mM EDTA and 2% FCS in HBSS) and incubated at 37°C, 200 rpm for amounts of time specified above each image panel. Tissues were not transferred into fresh EDTA solution throughout the duration of specified incubation time. Cell recovery did not substantially increase from 2 hr 35 min to either 4 hr 20 min or 6 hr 30 min incubations. This figure is not intended as a reference for an EDTA incubation time for optimal epithelial removal but to emphasize that individual optimization of methods for epithelial cell removal will be necessary based on technical and biological contexts of an experiment. EDTA (ethylenediamine tetraacetic acid); FCS (fetal calf serum); H&E (hematoxylin and eosin); HBSS (Hank’s balanced salt solution); hr (hour); min (minute); mL (milliliters); mM (millimolar); rpm (rotations per minute).

Further resolution of IELs into inferred functional subsets can be performed through flow cytometry, where a larger selection of antibodies is available for simultaneous cell labeling compared to *in situ* detection methods. Cell staining for flow cytometry requires cells to be dissociated from tissue into a single-cell suspension. Isolation of IELs relies on precise and accurate removal of only cells from the epithelial layer of intestinal tissues, so as not to obtain contaminating sub-epithelial cells that can confound biological interpretations. Epithelial cell removal is performed by incubating tissues in solution containing the chelating agent, ethylenediamine tetraacetic acid (EDTA), which dissociates epithelial cells from tissue, though protocols need to be catered to particular intestinal samples in different biological contexts. For example, in jejunum and ileum of 5-day-old pigs, incubation in EDTA solution for two sequential one hour incubations resulted in near complete epithelial removal from both crypts and villi, plus dissociation of a sizeable portion of non-epithelial cells (83). The same incubations performed on jejunum and ileum of 14-month-old pigs resulted in incomplete removal of epithelial crypt cells but less sub-epithelial contamination (83). Thus, biological context of intestinal samples influences which epithelial regions are removed and the degree of sub-epithelial contamination. Technical parameters must also be considered for efficient epithelial isolation. For example, duration of incubation in EDTA solution impacts epithelial recovery and purity. As seen in **Figure 4**, extended EDTA incubation times result in greater epithelial liberation but at the expense of increased potential for sub-epithelial contamination and alterations to cellular profiles associated with isolation procedures, including increased cell activation or death. Additional factors including epithelial integrity, epithelial surface area, tissue handling, tissue size, reagent volume, incubation temperatures, use of freshly prepared solutions, and EDTA concentrations must also be considered and optimized, as these variables can affect the duration of EDTA incubation required for efficient epithelial removal.

Since porcine ILC-IELs are only recently characterized *via* flow cytometry, most in-depth *ex vivo* phenotyping of porcine intestinal IELs pertains to T cell subsets, which we focus on for this review. While ILCs were identified as CD2^+^CD3ε^-^CD79α^-^CD172α^-^ cells in porcine intestine *via* flow cytometry (57), porcine T-IELs are more simply identified as CD3ε^+^ lymphocytes, and detection of additional phenotypic markers is implemented for in-depth phenotyping. Though a small fraction of CD4^+^ T cells are detected *via* flow cytometry in preparations of porcine IELs, *in situ* staining mentioned previously has validated CD4^+^ T cells are contaminating sub-epithelial cells and are thus not discussed further.

### IEL subsets: Phenotypes, inferred functions, and proportional distributions

Porcine IELs include major subsets of CD8 αβTCR T-IELs (further broken down into CD8αα^+^ and CD8αβ^+^ populations), γδ TCR T-IELs, and ILC-IELs. A summarization of major porcine intestinal IEL subsets and findings discussed in subsequent subsections is available in **Table 1**.

**Table 1:** Summary of major IEL subsets identified in porcine intestine.

IEL subset	Phenotypic identification	Locational distribution^*^	Key conclusions
CD8αα^+^ αβTCR T-IELs	Flow cytometry: CD3ε^+^γδTCR^-^CD4^-^CD8α^+^CD8β^-^ (67)	Small intestine: -/+ (67) Large intestine: + (67)	- Proposed as analogous to naturally-occurring, innate-like CD8αα^+^ αβTCR T-IELs identified in mice (1, 102–108) - Innate-like, effector functions speculated, including TCR-independent activation (67)
CD8αβ^+^ αβTCR T-IELs	Flow cytometry: CD3ε^+^γδTCR^-^CD4^-^CD8α^+^CD8β^+^ (67) RNA-ISH: *CD8B*+ (57)	Small intestine: +++ (67) Large intestine: ++ (67)	- Proposed as analogous to conventional, induced CD8αβ^+^ αβTCR T-IELs identified in mice and humans (109, 110) - Accumulate with age, likely dependent on antigen exposure (66, 67) - Activated in a TCR-dependent manner (111, 112), though capacity for TCR-independent activation is speculated (67)
γδTCR^+^ T-IELs	Flow cytometry: CD3ε^+^γδTCR^+^ (66, 67) RNA-ISH: *TRDC* ^+^ (57, 66)	Small intestine: + (66, 67) Large intestine: ++ (66, 67)	- Innate-like, effector functions speculated, including TCR-independent activation, phagocytosis, and antigen presentation (67, 111, 112)^ǂ^ - CD8α proposed as a marker for anti-inflammatory (CD8α^+^) versus pro-inflammatory (CD8α^-^) subsets (113)
ILC-IELs	Flow cytometry: CD2^+^CD3ε^-^CD79α^-^CD172α^-^ (57) Dual IF/RNA-ISH: *ITGAE* ^+^CD3ε^-^ (57)	Only identified in porcine ileum (57) Vastly outnumbered by *ITGAE* ^+^CD3ε^+^ T-IELs (57)	- Identified as group 1 ILCs that are distinct from NK cells (57) - Innate functions associated with cytotoxicity, tissue residency, and effector cells speculated (57, 79) - Higher proportional abundance in early life speculated (77, 79, 80, 83)^^^

^*^Values represent proportional rather than absolute abundances. -/+ <5% of total T-IELs; + 5-20% of total T-IELs; ++ 20-50% of total T-IELs; +++ >50% of total T-IELs.

^ǂ^Speculated capacity as phagocytes and antigen presenting cells based on co-expression of CD16 (an immunoglobulin receptor that facilitates phagocytosis) and MHC-II (an antigen presenting molecule) on a subset of γδ T-IELs shown in **Figure 5**.

^^^ILC-IELs not directly identified in research but instead inferred as CD2^+^CD4^-^CD8α^-^ IELs, which could encompass additional cell subsets.

IEL, intraepithelial lymphocyte; IF, immunofluorescence; ILC, innate lymphoid cell; ILC-IEL, intraepithelial innate lymphoid cell; NK, natural killer; RNA-ISH, RNA in situ hybridization; T-IEL, intraepithelial T lymphocyte; TCR, T cell receptor.

#### CD8 αβTCR T-IELs

In pigs, CD8 αβTCR T-IELs are labeled as CD3ε^+^γδTCR^-^CD4^-^ lymphocytes expressing CD8α and/or CD8β (67). In rodents, two subsets of CD8 αβTCR T-IELs exist: (1) ‘induced’ T-IELs expressing the traditional heterodimeric CD8αβ co-receptor and (2) ‘natural’ T-IELs expressing CD8αα homodimers. Induced CD8αβ^+^ αβTCR T-IELs are recruited to the intestine in an antigen-dependent manner by TCR-mediated antigen recognition (109, 110). Conversely, CD8αα^+^ αβTCR T-IELs occur naturally, migrating directly to the intestine from the thymus without TCR-mediated recruitment (102–104), and CD8αα acts as an inhibitory receptor rather than an activating TCR co-receptor (1, 105–108). In the small intestine of pigs, the majority of T-IELs are CD8 αβTCR T cells (γδTCR^-^CD4^-^CD8α^+^) and proportionally increase with age, whereas in the large intestine, only about half of T-IELs are CD8 αβTCR T cells (66). However, whether γδTCR^-^CD4^-^CD8α^+^ T-IELs expressed CD8β was not determined. We have since identified both CD8β^+^ and CD8β^-^ populations within γδTCR^-^CD4^-^CD8α^+^ T-IELs, indicating the presence of both innate-like CD8αα^+^ αβTCR T-IELs and traditional CD8αβ^+^ αβTCR T-IELs in pigs (67). Though the majority of porcine CD8α^+^ αβTCR T-IELs also express CD8β throughout the intestinal tract, CD8αα^+^ αβTCR T-IELs are found at lower frequencies (67). CD8αα^+^ αβTCR T-IELs have the highest proportional abundance in the large intestine (2-15% of T-IELs in cecum), are rare in the jejunum, and have intermediate proportions in the ileum (67). Moreover, porcine CD8αα^+^ αβTCR T-IELs have increased expression of CD16 [Fcγ receptor able to activate T cells independent of TCR engagement (114, 115)] and lower expression of CD27 [co-stimulatory receptor downregulated on effector and memory T cells (116, 117) and used as a marker of effector/memory T cells in pigs (118–124)] compared to porcine CD8αβ^+^ αβTCR T-IELs (67). Based on the limited phenotyping that has been performed, we speculate an innate-like, effector role for porcine CD8αα^+^ αβTCR T-IELs. CD8αα^+^ αβTCR T-IELs in pigs might be similar to natural as opposed to induced T-IELs described in other species, though further research is warranted to confirm or disprove. In further support of CD8αα^+^ αβTCR T-IELs being naturally-occurring T-IELs, others reported in review that CD4^-^CD8α^+^CD8β^-^ αβTCR T cells are some of the earliest T-IELs present in the porcine intestine, before antigen has largely been encountered (125), though we were unable to identify the original research these conclusions are derived from.

Though CD8αβ^+^ αβTCR T cells are traditionally considered conventional T cells that rely on TCR-mediated activation, we speculate functional properties of CD8αβ^+^ αβTCR T-IELs is influenced by intestinal location of cells being assessed. Increased proportions of CD8αβ^+^ αβTCR T-IELs express CD16 in the cecum compared to jejunum and ileum, albeit at lower levels than that of cecal CD8αα^+^ αβTCR T-IELs [15-55% of cecal CD8αβ^+^ αβTCR T-IELs versus 35-80% of cecal CD8αα^+^ αβTCR T-IELs (67)]. CD16 expression on a subset of large intestinal CD8αβ^+^ αβTCR T-IELs therefore suggests at least some CD8αβ^+^ αβTCR T-IELs might also have innate-like functions. However, no functional studies of CD8αβ^+^ αβTCR T-IELs have yet been conducted in the porcine large intestine to our knowledge.

CD8αβ^+^ αβTCR T-IELs from porcine small intestine have been isolated as CD8β^+^ cells and studied at a functional level *in vitro*. NKG2D is an NK activating receptor that recognizes major histocompatibility complex (MHC) class I-related molecules (MICs) expressed by stressed epithelial cells. Activation of NKG2D initiates TCR-independent cell activation (126) or costimulation to amplify TCR-mediated T cell activation and augment cellular activation and cytotoxicity (127–129).While small intestinal CD8β^+^ IELs in pigs have only moderate expression of *KLRK1* (encoding NKG2D), *KLRK1* expression is increased more than two-fold with anti-CD3ε stimulation (111, 112). Thus, small intestinal CD8αβ^+^ αβTCR T-IELs are activated in an adaptive, TCR-dependent manner (via anti-CD3ε stimulation). Moreover, anti-CD3ε stimulation of peripheral CD8β^+^ T cells and γδTCR^+^ IELs (γδ T-IELs; from similar small intestinal locations as CD8β^+^ IELs) did not result in increased *KLRK1* expression (112), indicating TCR-dependent NKG2D upregulation is not generalized across anatomical locations or T cell lineages. Therefore, CD8αβ^+^ αβTCR T-IELs mirror induced IELs in their patterns of TCR-mediated activation but also had different activation dynamics compared to peripheral CD8β^+^ T cells. The use of single-cell RNA sequencing (scRNA-seq) to compare CD8αβ^+^ αβTCR T cells from porcine ileum and blood further supports the notion of functional distinctions, as intestinal CD8αβ^+^ αβTCR T cells are transcriptionally distinct from cells in the blood (57).

#### γδTCR T-IELs

Due to previous inabilities for *in situ* detection of γδTCR^+^ T cells described earlier, knowledge of γδTCR T-IEL distributions is derived from flow cytometry data that reports only proportional rather than absolute cell abundances. The proportion of γδTCR^+^ within total T-IELs generally increases from proximal to distal small intestine and from small to large intestine, with similar proportions in both the cecum and colon of the large intestine (66). Similar to γδTCR T cells in circulation (58, 124, 130–133), there are decreased proportions of small intestinal γδTCR T-IELs as pigs age (66), presumably due to recruitment/expansion primarily of CD8αβ^+^ αβTCR T-IELs that mirror descriptions of induced T-IELs in humans and mice (109, 110). Thus, though it is unknown whether absolute numbers of γδTCR T-IELs are altered with age (due to lack of *in situ* quantification), proportions of γδTCR T-IELs decrease with age at the expense of increasing proportions of other T-IELs.

**Figure 5:**
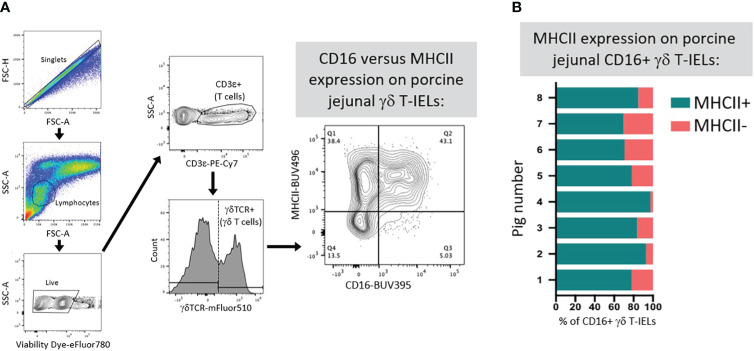
Co-expression of CD16 and MHC-II on γδ T-IELs in porcine jejunum. **(A)** Representative flow cytometry gating showing the expression of CD16 and MHC-II on gated γδ T-IELs in epithelial-enriched cell fractions from jejunum of a 6-week-old pig. **(B)** Stacked bar plot of the percentage of CD16^+^ γδ T-IELs that are MHC-II^+^ (turquoise) versus MHC-II^-^ (salmon). Data are derived from jejunum of eight 6-week-old pigs. Epithelial-enriched cell fractions were isolated from jejunum and stained for flow cytometry as previously described (67). Detection reagents used for flow cytometry staining are as follows: Fixable Viability Dye eFluor780 (ThermoFisher Scientific 65-0865-14); anti-CD3ε-PE-Cy7 (clone BB23-8E6-8C8; BD 561477); anti-γδTCR (clone PGBL22A; Washington State University PG2032; conjugated to mFluor510 fluorophore by Caprico Biotechnologies); anti-CD16 (clone G7; BioRad MCA1971GA) detected with anti-mouse IgG1 (clone A85-1; BD 740234); MHC-II (clone 2E9/13; BioRad MCA2314GA) detected with anti-mouse IgG2b-BUV496 (clone R12-3; BD 750517). FSC-A (forward-scatter area); FSC-H (forward-scatter height); Ig (immunoglobulin); MHC (major histocompatibility complex); SSC-A (side-scatter area); T-IEL (intraepithelial T lymphocyte); TCR (T cell receptor).

Limited functional studies of intestinal γδTCR T-IELs in pigs indicate innate-like roles. γδTCR^+^ IELs have higher expression of *KLRK1* (encoding NKG2D, functionally described in previous section) than do CD8β^+^ IELs, indicating their enhanced ability to mediate epithelial stress signals by recognizing MICs on IECs (111, 112). Moreover, more porcine γδTCR T-IELs express CD16 than do CD8β^+^ IELs (67). In humans, CD16 on γδTCR T cells binds to opsonizing antibodies to facilitate phagocytosis, followed by presentation of ingested antigen on MHC-II molecules (134–136). It’s plausible that porcine γδTCR T-IELs might have similar capacities for CD16-mediated phagocytosis and antigen presentation, as circulating γδTCR T cells can act as antigen presenting cells in pigs (137). Most CD16^+^ γδTCR T-IELs are also MHC-II^+^ in the porcine jejunum (**Figures 5A, B**), supporting the idea of dual ability to phagocytose and present antigens. Compared to CD8β^+^ IELs, porcine γδTCR^+^ IELs are more susceptible to Salmonella infection *in vitro*, indicating γδTCR T-IELs as a potential target for pathogenic infection (138), perhaps related to phagocytic abilities of γδTCR T-IELs.

CD8α expression in the absence of CD8β (presumably expressed as CD8αα homodimers) is considered a marker of differentiation for CD4 αβTCR T cells in pigs (121, 139–145) but may also serve as a marker to differentiate porcine γδTCR T-IEL populations and functions. Most γδTCR T-IELs are CD2^+^CD8α^+^ (presumably expressed as CD8αα homodimers), with a smaller proportion of CD2^+^CD8α^-^ γδTCR T-IELs primarily located in the proximal small intestine (66, 67). Fewer porcine CD2^+^CD8α^-^ γδTCR T-IELs express CD27 compared to CD2^+^CD8α^+^ γδTCR T-IELs, indicating a potential difference in effector capacity between the two γδTCR T-IEL subsets (66, 67). Thus, CD8α expression by CD2^+^ γδTCR T-IELs likely correlates with altered functional capacities, though these functional differences are incompletely understood. Comparison of porcine CD2^+^CD8α^-^ and CD2^+^CD8α^+^ γδTCR T cells completed outside of the context of the intestine supports CD8α as a marker gained upon activation/differentiation (120, 123, 124, 132, 137, 146–151). However, we are cautious to apply such findings to γδTCR T-IELs, as IELs represent a unique compartment of immune cells that may have different functional dynamics. In other work directly applied to γδTCR T-IELs in porcine ileum, CD2^+^CD8α^+^ γδTCR T-IELs were proposed as anti-inflammatory and CD2^+^CD8α^-^ γδTCR T-IELs as pro-inflammatory mediators, albeit findings result from studies in gnotobiotic pigs infected with human rotavirus (113). Interestingly, findings draw parallels to documented inhibitory functions of CD8αα homodimers expressed by IELs of other species (1, 105–108). Wen et al. (113) describe higher frequencies of Foxp3^+^ cells within CD2^+^CD8α^+^ γδTCR T-IELs in comparison to both CD2^+^CD8α^-^ γδTCR T-IELs and circulating CD2^+^CD8α^+^ γδTCR T cells, indicating enrichment of regulatory T cells within the CD2^+^CD8α^+^ γδTCR T-IEL compartment, though percentages were consistently low (<0.4%). When cultured, CD2^+^CD8α^+^ γδTCR T-IELs had greater spontaneous expression of *IL10* mRNA. Following stimulation with the phosphoantigen, isopentenyl pyrophosphate, in combination with IL-2, greater expression of *IL10* was induced in CD2^+^CD8α^+^ γδTCR T-IELs, and greater expression of *IFNG* was induced in CD2^+^CD8α^-^ γδTCR T-IELs. Thus, analysis of *IL10* and *IFNG* expression indicated propensity of anti-inflammatory cytokine production in CD2^+^CD8α^+^ γδTCR T-IELs and pro-inflammatory cytokine production in CD2^+^CD8α^-^ γδTCR T-IELs (113). Therefore, results bring forward the question of whether CD8α expression might be associated with pro- versus anti-inflammatory bias in γδTCR T-IELs, but again, whether results are applicable to conventional pigs remains unknown. As CD2^+^CD8α^-^ γδTCR T-IELs with inferred pro-inflammatory capacity are preferentially located in the proximal small intestine and have decreased expression of CD27 in conventional pigs (66, 67), we speculate γδ T-IEL responses are compartmentalized by intestinal region.

#### ILC-IELs

Current knowledge of intestinal ILC-IELs in pigs is severely limited due to their very recent discovery (57). However, study of ILC-IELs in the porcine intestine is an exciting new research frontier, as methods for their identification *via in situ* and *ex vivo* staining are now available (57). ILC-IELs are identified as group 1 ILCs in pigs (57), which encompasses both NK cells and ILC1s by classical definitions. However, porcine intestinal ILC-IELs are difficult to discretely classify as NK cells or ILC1s due to tissue-specific characteristics, and this remains an issue even in humans and rodents (15, 152–155). Porcine NK cells are CD3ε^-^CD2^+^CD8α^+^ cells (55, 58, 142, 156, 157) and are scarce throughout the porcine intestine (62, 82, 158–161). However, group 1 ILCs in porcine ileum are transcriptionally distinct from porcine peripheral NK cells, including lack of *CD8A* expression, the gene encoding for CD8α and used to identify porcine NK cells (57). Group 1 ILCs located in the ileal epithelium instead have gene expression profiles supporting tissue residency, cell activation, and altered metabolic states compared to peripheral NK cells. The majority of ileal group 1 ILCs have transcriptional profiles supporting cytotoxic functions, while smaller subsets express genes indicating cellular division/replication or other effector cell profiles that is characterized by high expression of activation-associated genes, including *CTSW*, *XCL1, CCR9*, and genes encoding MHC-II (57). Recent work described above provides only the first established evidence of non-NK intestinal ILC-IELs in pigs, and further work assessing phenotype, function, and distributions is warranted to understand their biological functions.

Though research on porcine intestinal ILC-IELs is only recently emerging, we can cautiously extrapolate ILC-IEL functions from past studies. Previous research hints at the potential presence of ILCs as CD3ε^-^CD2^+^CD8α^-^ cells in the pig intestinal tract, which excludes both T cell (CD3ε^+^) and NK cell (CD3ε^-^CD2^+^CD8α^+^) phenotypes. Shortly after birth, most CD2^+^ cells in the porcine intestine are CD4^-^CD8α^-^ (77, 79, 80, 83) and might represent naturally-occurring populations of ILCs that predominate in the intestinal tract prior to antigen-dependent lymphocyte recruitment/expansion. Most but not all CD2^+^ staining in small intestinal epithelium colocalizes with CD3ε^+^ staining (68), giving another potential indication of a minority of intestinal CD3ε^-^CD2^+^ ILCs in pigs. Within the duodenal and ileal epithelium, IELs located near the basement membrane are primarily CD2^+^CD4^-^CD8α^+^ (likely NK cells or T cells) and morphologically consistent with small, resting lymphocytes; however, IELs located in epithelial nuclear and apical zones that are in closer proximity to the lumen are mostly CD2^+^CD4^-^CD8α^-^ and have enlarged cytoplasm with granules and ribosomes (79), thus leaving the possibility for existence of intraepithelial ILCs in epithelial nuclear and apical zones that act as first-line effector cells with cytotoxic capabilities at epithelial barriers. Though the CD3ε^-^CD2^+^ phenotype excludes T cells and the CD2^+^CD4^-^CD8α^-^ phenotype excludes NK cells, expression of specific NK, B, or myeloid lineage leukocyte markers was not fully assessed in a single study, making it impossible to definitively discern the cell lineage of either CD3ε^-^CD2^+^ or CD2^+^CD4^-^CD8α^-^ cells. Thus, recent identification of porcine intestinal ILCs and development of tools for their detection present exciting opportunities for future research to better understand biological importance of non-NK ILCs in pigs, including intestinal ILC-IELs.

### Indications of IEL effector functions and tissue residency

Tissue resident lymphocytes represent effector immune cells found at local, non-lymphoid tissue sites and can act as first-line immune responders, thus comprising an important component of immune defense often targeted *via* immunomodulatory strategies [reviewed in (70, 162–164)]. Without deciphering between particular IEL subsets, several studies have indicated IELs are resident, effector cells in the porcine small intestine. One indicator of tissue residency for porcine intestinal IELs is expression of *ITGAE* (57), which encodes for a subunit of CD103, a marker of tissue residency in humans and rodents (70). Another indicator of tissue residency for porcine intestinal IELs is an observed low exchange rate of IELs with lymphocytes recirculating through the lymph (165, 166), suggesting IELs are largely self-replenishing/long-lived resident cells, at least under steady state conditions. IEL self-replenishment is supported by observing most bromodeoxyuridine (BrdU)^+^CD2^+^ cells (indicating proliferating lymphocytes) in the intestinal tract are intraepithelial (65, 83).

Molecular characterization and spatial context of IELs also provides relevant functional insight. About half of porcine small intestinal IELs contain cytoplasmic granules accompanied by prominent mitochondria, ribosomes, nuclei, and nucleoli (167) that are indicative of cytotoxic capacity and elevated metabolic/protein production. IELs with enlarged cytoplasm, granules, and prominent organelles are preferentially found in apical epithelial locations in closer proximity to the lumen, whereas the majority of total IELs are located in the basement membrane, do not have enlarged cytoplasm, and have dense, round nuclei (79, 97). From these observations, we speculate a correlation between luminal proximity and IEL effector function, where IELs in the basement membrane are naïve or patrolling IELs that require additional activating signals to infiltrate the epithelium. In support, inoculating isolated porcine jejunal loops with porcine epidemic diarrhea virus (PEDV) or *Bacillus subtilis* resulted in overall increases in IELs. Specifically, a proportional increase of apically-located IELs and an increase in proliferating IELs that were mostly apically located occurred after inoculation (87), indicating both pathogenic and non-pathogenic microbial cues derived from the lumen can shape IEL responses associated with their localization.

Though IEL responses to infectious challenge are not a focus of this review, we discuss the recent work by Li and colleagues (87) in greater detail to conclude this section due to implications on porcine IEL immunosurveillance, migratory behaviors, and effector functions. Li et al. recently studied the dynamics of isolated small intestinal porcine IELs in co-culture systems with epithelial cell lines in the context of PEDV infection and pre-exposure. Treatment of co-cultures with live PEDV resulted in enhanced migration of IELs across the epithelial monolayer, which was inhibited in the presence of a CCL2 receptor inhibitor. As CCL2 protein expression was mainly concentrated to the apical epithelial membrane, it appears that IEL migratory responsiveness is reliant on epithelial-IEL crosstalk, by which IELs follow a CCL2 chemotactic gradient to gain closer proximity to the lumen, all without deterioration to the epithelial barrier. Moreover, treatment of PEDV-infected epithelial cells with IELs pre-stimulated with whole-inactivated PEDV resulted in decreased viral titers and increased IEL cytotoxicity, perforin expression, IFN-γ expression, STAT1 phosphorylation, interferon-stimulated gene (ISG) mRNA expression, and targeted apoptosis of virally-infected epithelial cells. Lastly, the ability of IELs to exert effector functions contributing to anti-viral immunity was dependent on IFN-γ activity but was independent of direct cell-cell contact, perforin, and granzymes (87). Collectively, results emphasize the importance of cell contact-independent activation of IELs resulting in migration towards epithelial-derived chemotactic signals at the apical epithelium surface. IEL activation also results in exertion of effector functions including proliferation, targeted killing of infected (and perhaps stressed) epithelial cells, cytotoxicity, and cytokine production. Interestingly, trans-epithelial migration of porcine IELs mediated by IEC-derived signaling in response to microbial stimuli mirrors IEL surveillance and migration dynamics observed in mice (7, 23). Effector functions including porcine IEL cytotoxicity, antiviral activity, and cytokine production (TGF-β) are also reported for porcine intestinal IELs (167), although outside of the context of epithelial co-cultures systems. While TCR-mediated activation (anti-CD3ε stimulation) did not induce an anti-viral response in work by Li et al. (87), TCR stimulation can induce changes in expression of activating receptor NKG2D on subsets of porcine IELs [discussed in earlier sections (112)]. Different anti-CD3ε clones were used to stimulate cells across studies, and anti-CD3ε antibodies used by Li et al. were not verified to activate T cells *via* TCR stimulation. Overall, further investigation is required to better understand dynamics of porcine intestinal IEL activation that occurs with or without TCR stimulation and how these dynamics tie into IEL roles of immunosurveillance, migratory behaviors, and effector functions in different biological contexts.

### Antigen exposure influences IEL recruitment and maturation

From the preceding section, we can gather porcine intestinal IELs have functional roles as tissue resident, effector cells. However, IEL maturation into functionally capable cells is dependent on antigen exposure, as discussed in this section. In the next three subsections, we discuss how microbial, dietary/oral, and environmental antigen exposures affect T-IEL maturation in the developing intestinal immune system.

#### Microbial colonization

The influence of microbial exposure on intestinal IEL development in pigs is best demonstrated by comparing germ-free (GF) and conventional animals. Intestinal IEL communities in GF pigs resemble those of very young animals in terms of both cell quantities and phenotypes (65, 80, 83), indicating intestinal IEL maturation is dependent on microbial exposure accrued with age when reared in a conventional environment. GF pigs have fewer CD2^+^ lymphocytes present in the intestinal tract, including less than one-third as many IELs in ileum or jejunum of GF versus conventional pigs aged one to two months old (65, 83). A large percentage of CD2^+^ cells remain CD4^-^CD8α^-^ in the epithelium of GF compared to conventional, age-matched animals (80, 83, 168), similar to high frequencies of CD2^+^CD4^-^CD8α^-^ IELs in young animals. Context of colonization also influences IEL abundances in the intestinal tract, as the number of IELs in the jejunum and ileum of specific pathogen-free (SPF) animals is intermediate compared to high IEL numbers in conventional pigs and low IEL numbers in GF pigs (65). Thus, intestinal IEL abundance and phenotype in pigs depends on both the presence and context of microbial colonization.

It also remains likely that proximity to luminal microbiota influences effector potential of intestinal IELs. As mentioned in preceding sections, IELs preferentially congregate to intestinal villi that are more intimately associated with the lumen over intestinal crypts that provide a more shielded niche (65). IELs located closer to the lumen have enlarged cytoplasm containing granules, ribosomes, and mitochondria, consistent with increased effector functions (79), and IELs migrate across the epithelium in response to pathogenic and non-pathogenic microbial stimuli (87).

#### Dietary/oral antigen

Close proximity of IELs with the intestinal lumen likely result in intimate interactions with dietary/orally-administered antigens that also shape functions and behaviors of IELs. For example, seven-day-old pigs given dietary nutrients intravenously to bypass the intestinal tract (total parenteral nutrition [TPN]) have nearly three-fold fewer IELs per villus in jejunum compared to conventionally-fed animals (169), indicating exposure to dietary antigens at the intestine-lumen interface is critical for IEL recruitment/expansion.

Increases in IEL numbers are generally associated with enhanced immune activation of IELs, while decreased IEL numbers are associated with reduced immune activation. For example, pigs fed bacteria producing the immune enhancer, 4,4’-diaponeurosporene, have increased IEL numbers in ileum (170), while 57% fewer jejunal IELs are observed in pigs fed the immunosuppressor, dexamethasone (171), compared to control animals. Though IEL abundances in the intestine are the primary readout for potential immunomodulatory effects of dietary additives, phenotypic and functional studies of porcine IELs in response to dietary immunomodulators are hugely lacking. Thus, IEL abundances cannot be linked directly to any stimulated or suppressed behaviors and are probably largely dependent on more specific biological context, such as pig age, environment, health status, and potential mechanistic outcomes of intervention strategies being implemented. Regardless, we summarize our current knowledge on the effects a subset of dietary immunomodulators have on porcine IEL abundances in the intestinal tract. However, we again emphasize the need for deeper phenotypic and functional analyses of IELs in future research.

Recruitment and maturation of intestinal IELs is largely dependent on the microbiota, and dietary additives affecting the microbiota, such as probiotics and prebiotics, also exert immunomodulatory effects on porcine intestinal IELs. Oral administration of various probiotics leads to increased numbers of IELs in the small and large intestine (86, 97, 101, 172, 173), including increased numbers of CD3ε^+^CD8α^+^ cells (174) that might represent induced T-IELs recruited to the intestinal tract. Feeding prebiotics can also have similar effects on intestinal IELs, where feeding rice bran (175), yeast products (95), plant-derived polysaccharides (173), and chito-oligosaccharides (176) results in increased numbers of IELs in the porcine small and/or large intestine. However, yeast product and chito-oligosaccharide feeding causes increases in IELs of the proximal small intestine (duodenum, jejunum) but decreases in distal small intestine [ileum (95, 176)], suggesting regionally-specific immunomodulatory roles. Other dietary additives decrease the number of IELs in the small and/or large intestine of pigs. Plant extracts decrease IEL numbers in jejunum but not ileum or colon (88, 90), whereas spray-dried porcine plasma decreases IEL numbers in jejunum and colon (89, 90).

Oral antibiotic administration has variable effects on IEL numbers. Avilamycin, olaquindox, and cyadox administration result in decreased small intestinal IEL numbers (88, 92). However, others have reported increased numbers of IELs in small intestine after feeding chlorotetracycline (173) or a combination of chlorotetracycline, sulfamethazine, and penicillin (175). Thus, the effect of oral antibiotic administration on IELs is probably dependent on the specific antibiotic being used and the intestinal region being assessed.

Recent limitations on the use of antibiotics as growth promoters has resulted in the need to identify dietary alternatives that may not only promote animal growth but also improve gut health through modulation of the intestinal immune system. As IELs at the epithelial-lumen interface are in close contact with dietary substances and the microbiota, IELs are a prime target to study for the effects of dietary amendment with antibiotic alternatives. As demonstrated above, many dietary additives affect the intestinal microbiota, which likely leads to production of microbial products that contribute to pig intestinal and overall health outcomes. Therefore, understanding the mode of action for various dietary immunomodulators on intestinal health is crucial for developing mechanistic understandings that can be applied through targeted approaches for improving pig health in the future.

#### Environment

Environmental hygiene influences development of intestinal IELs. Weaned pigs transported to separate facilities have greater numbers of small intestinal IELs and earlier expansion of CD8α^+^ and γδTCR^+^ IELs compared to weaned pigs reared on the same farm (177, 178). Though other variables associated with transport cannot be excluded, results suggest IEL recruitment, expansion, and/or maturation in the post-weaning period is accelerated by diverse antigen exposure encountered upon introduction to new environments.

### IEL dynamics associated with age and weaning

Weaning is a major stressor that occurs in early life for pigs (~3 or 4 weeks of age), prior to complete development of the intestinal immune system [5-8 weeks of age (46, 77, 80, 125)]. Industry-standard weaning occurs at ~21 days of life in the United States and includes removal of piglets from the sow, introduction to a new environment, abrupt conversion to a solid food diet, intermingling with non-littermates, and often transportation over long distances. We collectively refer to all of these events as ‘weaning’ for the rest of this review. Weaning results in stress and intestinal distress characterized by a combination of inflammation, diarrhea, disease susceptibility, weight loss, increased epithelial permeability, and decreased nutrient absorption (47, 48). Thus, conventional pig weaning represents a major early-life stressor that is detrimental to intestinal health and may impact subsequent intestinal immune system development and intestinal immune cell maturation. Weaning pigs at an earlier age can exacerbate negative weaning impacts and also affects intestinal IELs. Weaning is generally associated with a post-weaning increase in IEL abundances of both small and large intestine that is most notable in the first few days after weaning (76, 77, 81, 93, 97, 179–181). However, detailed phenotypic analyses of IELs in age-matched weaned and non-weaned animals are extremely limited.

Though intestinal IEL populations expand in weaned pigs, full capacity to mount immune responses geared towards intestinal defense or tolerance are not achieved until a later age (46). IELs from pigs weaned at 21 days of age undergo metabolic shifts that increase substrate metabolism and adenosine triphosphate (ATP) production within the first week post-weaning, followed by resuming lower baseline values as post-weaning time increases (182). Moreover, IELs from pigs weaned at 21 days of age cannot proliferate in response to stimulation with T cell mitogens, phytohaemagglutinin (PHA) or concavalin A (ConA), until 11 weeks of age (183). Results suggest an enhanced metabolic state for porcine intestinal IELs but limited effector response shortly after weaning, as the intestinal immune system is not fully developed and IELs continue to mature. Though weaning accelerates the recruitment/expansion of intestinal IELs, as indicated by their increased frequencies in weaned versus non-weaned pigs, weaning may delay the ability of IELs to form an appropriate effector response. As mentioned above, IELs from pigs weaned at 21 days of age do not respond to *in vitro* PHA or ConA stimulation until 11 weeks of age (183). In contrast, IELs from pigs that are not weaned are able to proliferate in response to PHA or ConA starting at 8 weeks of age (183), indicating weaning delayed the ability of intestinal IELs to mount an effector response.

Results in the preceding paragraph are from functional study of IELs derived from small intestine of pigs, and functions of large intestinal IELs are unresolved in pigs. Our group has assessed expression of CD27 by T-IELs derived from both small and large intestine at one, three, and five weeks post-weaning (66), which can lend inference into functional capacities of T-IELs. A similar majority percentage of CD4^-^CD8α^+^ αβTCR, CD2^+^CD8α^+^ γδTCR, and CD2^+^CD8α^-^ γδTCR T-IELs (making up the majority of total T-IELs) express CD27 one week post-weaning in jejunum, ileum, cecum, and colon. However, the percentage of CD27^+^ cells significantly drops in ileum and to a more drastic extent in cecum and colon by three weeks post-weaning while staying constant in the jejunum (66). By using loss of CD27 as an inferred readout of increased T cell activation, results suggest T-IELs in the large intestine and distal small intestine may be activated at an earlier age. Functional studies in the preceding paragraph were executed using IELs from jejunum (182) and an unspecified small intestinal location (183). Lack of study for T-IELs from the distal intestinal tract, particularly the large intestine, leaves the question of whether IEL activation and ability to mount immune responses might be achieved at an earlier post-weaning age at alternate intestinal locations. Moreover, proportions of different T-IEL subsets (primarily CD4^-^CD8α^+^CD8β^+^ αβTCR, CD4^-^CD8α^+^CD8β^-^ αβTCR, CD2^+^CD8α^+^ γδTCR, and CD2^+^CD8α^-^ γδTCR T-IELs) vary by time post-weaning across different intestinal location and have different inferred functional capacities (66, 67), again emphasizing the importance of considering locational context of IELs used in previous functional studies.

Though IEL populations begin to rapidly expand in the porcine small intestine within the first few weeks of life in accordance with antigen exposure, CD8α^+^ IELs don’t begin appearing with regular frequency until around five weeks of age, two weeks into the post-weaning period for pigs weaned at 3 weeks of age (77). As the post-weaning period provides exposure to new antigens, it’s possible that the majority of CD8α^+^ IELs are recruited to the intestinal epithelium in response to increased antigen exposure that occurs after weaning. In preceding sections, we established the majority of CD8α^+^ IELs in the porcine small intestine are CD8αβ^+^ αβTCR T-IELs, and CD8αβ^+^ αβTCR T-IELs proportionally increase to make up the largest majority of porcine intestinal T-IELs with post-weaning age (66, 67). In mice, CD8αβ^+^ αβTCR T-IELs are termed induced IELs because they are recruited to the intestinal epithelium in response to age-associated, antigen-specific activation (184–187). Thus, it’s possible that similar phenomena occur in the pig post-weaning period, where post-weaning expansion of the IEL community is largely due to antigen-specific recruitment of CD8αβ^+^ αβTCR T-IELs.

Collectively, weaning and age-associated changes in IELs of the porcine intestine are intercalated with events facilitating antigen exposure, including microbial, environmental, and dietary/oral antigen exposure described above. As we can now appreciate, intestinal IELs take time to mature and are influenced by the factors of the developing intestinal tract in which they reside. Thus, results show the importance of stressful early life events and antigenic exposure in shaping the intestinal immune system, including that of porcine intestinal IELs.

### Pigs as biomedical models for intestinal IEL research

Throughout previous sections, porcine IEL cross-species parallels were mainly drawn to mice, largely because mice are the primary model species from which IELs are studied. However, major differences between humans and mice exist (discussed below), indicating mice may be a suboptimal model for biomedical intestinal IEL research. Compared to rodents, pigs are promising biomedical models for intestinal research due to greater intestinal physiological, nutritional, and microbial similarities to humans (186–192). Comparability of the intestinal immune system in pigs and humans still requires further investigation to understand key early life differences, including placental transfer of maternal immunity present in humans and mice but not pigs (42). However, due to lack of placentally-transferred maternal immunity, pigs serve as an excellent model to study the impact of maternal immune transfer on subsequent immune system development due to the ability to derive agammaglobulinemic animals. Overall, present knowledge of porcine intestinal IELs presented in this review gives initial indication for suitability of pigs as biomedical models for intestinal IEL research, as summarized in **Table 2** and discussed below.

**Table 2:** Advantages and drawbacks on utility of pigs as biomedical models for intestinal IEL research.

Advantages
- Human intestinal physiology, nutrition, and microbiota more similar to pigs than mice (188–194) - Proportions of γδTCR T-IELs, CD8αβ^+^ αβTCR T-IELs, and CD8αα^+^ αβTCR T-IELs in human intestine more similar to pigs than mice (1, 7, 25, 29, 66, 67, 195–197) - Transcriptional similarities between human and porcine intestinal immune and epithelial cells identified *via* high-resolution transcriptomics (57, 198–200) - Biomedical applications using pig models have cross-utility to improve pig health and global food security
Drawbacks
- Lack of placental transfer in pigs but not humans or mice may alter intestinal development/IEL maturation trajectories (42) - CD4 αβTCR T-IELs present in humans and mice but are absent/rare in pigs (7, 25, 76–80, 195–197, 201) - Immunoreagent toolbox more limited in pigs than mice and humans

IEL, intraepithelial lymphocyte; T-IEL, intraepithelial T lymphocyte; TCR, T cell receptor.

Immune responses and health outcomes are results of culminating signals derived from the overall cellular community of a tissue (202–207). Thus, the proportional composition of cellular constituents is a major factor to consider when selecting a biomedical model to accurately recapitulate human biological systems. Proportions of specific intestinal T-IEL subsets are vastly different between humans and mice while being more similar to pigs in several ways. In the small intestine, over half of human T-IELs are CD8αβ^+^ αβTCR T-IELs [γδTCR^-^αβTCR^+^CD4^-^CD8α^+^CD8β^+^ (25)], whereas most small intestinal T-IELs in mice are instead γδTCR T-IELs [γδTCR^+^ (7, 25, 195–197)]. Similar to humans, most porcine T-IELs are CD8αβ^+^ αβTCR T-IELs in the small intestine [γδTCR^-^CD4^-^CD8α^+^CD8β^+^ (67)]. In humans, the proportion of γδTCR T-IELs (γδTCR^+^) increases from proximal to distal intestine, whereas γδTCR T-IEL proportions have a proximal-to-distal decline in mice (7, 25, 195–197). Again similar to humans, porcine γδTCR T-IELs (γδTCR^+^) make up increasing proportions of total intestinal T-IELs along a proximal-to-distal gradient (66, 67). Another key difference in cellular compositions of T-IELs in human and mice is the presence of CD8αα^+^ αβTCR T-IELs (γδTCR^-^αβTCR^+^CD4^-^CD8α^+^CD8β^-^), which make up ~20-40% of total T-IELs in both small and large intestine of mice (7, 25). In contrast, CD8αα^+^ αβTCR T-IELs are extremely rare or absent from humans (1, 25, 29). Though CD8αα^+^ αβTCR T-IELs (γδTCR^-^CD4^-^CD8α^+^CD8β^-^) were recently discovered in the porcine intestinal tract, CD8αα^+^ αβTCR T-IELs make up only a small fraction of small intestinal (<5%) and large intestinal (average 5-10%) T-IELs (67), thus representing a rare T-IEL subset in pigs more closely mirroring compositional proportions in humans than mice. Though T-IEL compositions between humans and pigs are more similar than human-to-mouse comparisons regarding γδTCR, CD8αβ^+^ αβTCR, and CD8αα^+^ αβTCR T-IEL proportions, differences in human versus pig T-IEL compositions do arise, largely from the presence of CD4 αβTCR T-IELs in humans (γδTCR^-^αβTCR^+^CD4^+^) but not pigs. While up to 20% of small intestinal and ~35-45% of large intestinal T-IELs are CD4^+^ in humans (25, 201), CD4 αβTCR T-IELs have been validated through *in situ* staining to be absent or extremely rare in porcine intestinal epithelium (addressed in preceding sections). However, similar to pigs (addressed in preceding sections) some human CD4 αβTCR T cells identified in epithelial cell fractions *via ex vivo* phenotyping could be contaminating sub-epithelial cells. Collectively, community compositions of IELs in pigs and humans share proportional similarities, especially in the small intestine where IELs are more abundant and the majority of IEL research is conducted. Therefore, pigs may be more suitable for the study of intestinal T-IELs than mice, as the overall cellular community from which culminating signals are derived are similar in regards to proportions of γδTCR T-IELs, CD8αβ^+^ αβTCR T-IELs, and CD8αα^+^ αβTCR T-IELs; however, the absence of CD4 αβTCR T-IELs in pigs provides a limitation for modeling intestinal CD4 αβTCR T-IELs found in human intestinal epithelium.

In addition to phenotyping a limited number of cellular markers at the protein level to identify proportions of specific IEL populations, high-resolution transcriptional studies suggest pig-to-human similarities of intestinal cells. A recent scRNA-seq study identified conserved expression of several cell type-canonical genes between pig and human epithelial and immune cells that were sparsely expressed in mice (198). An additional scRNA-seq study defining lymphocyte subsets (including T cells and ILCs) at enhanced resolution also identified transcriptional similarities, similar localization patterns, and conserved inferred functions of lymphocytes from porcine and human ileum (57). Yet another scRNA-seq study highlights conserved functions and compositions of epithelial cells between human and pig ileum; however, compositional similarities should be interpreted with caution due to vast differences in cell isolation procedures used between species (199). One final scRNA-seq study expanded analysis to assess transcriptomes of epithelial cells in duodenum, jejunum, and ileum of the porcine small intestine, finding again transcriptional similarities between humans and pigs for the majority of epithelial cells (200). Thus, high-resolution transcriptomic analyses support transcriptional conservation between immune and epithelial cells of human and porcine intestine, but scRNA-seq studies have so far been limited to only the small intestine. Therefore, further research is warranted to expand understanding of potential regional specialization and/or species overlap at additional intestinal locations. No scRNA-seq study has explicitly differentiated between intraepithelial and subepithelial lymphocytes in pigs, as the exclusive study of IELs is complicated by necessity of epithelial cell isolation that is plagued by contamination with sub-epithelial cells (discussed in preceding sections), though this is a species-agnostic issue. Regardless, high-resolution transcriptional profiling has revealed indications of transcriptional conservation across intestinal cells in humans and pigs, which encompasses IELs.

Altogether, preliminary phenotypic research at the protein and gene level indicates porcine IELs more closely mirror human IELs than do IELs from mice. Though immunoreagent availability in pigs is more limited than mice or humans, the toolbox of porcine-specific immunoreagents is rapidly expanding. Breakthroughs in fundamental porcine immunology and intestinal biology are also constantly occurring, leaving the study of porcine IELs, including both T-IELs and ILC-IELs, as an exciting, emerging research frontier. Besides phenotypic descriptions of pigs that draw parallels to humans, functional studies mentioned in preceding sections also highlight the versatility of pigs to study IEL functions that might more closely recapitulate human biology, though further investigation is still required. Unlike most model species, biomedical applications of pigs for intestinal IEL research falls under the One Health initiative, as biomedical research utilizing porcine models has cross-utility for improving pig health related to global food security, a growing world-wide crisis (208). Further research into porcine intestinal IEL functions is thus advantageous for multiple realms of research, emphasizing an integrated research approach producing applicational duality for human and animal health.

## Author contributions

JW wrote the manuscript and performed experiments. CL edited the manuscript. All authors contributed to the article and approved the submitted version.
